# Evaluation of Antioxidant and Anti-Melanogenic Activity of Different Extracts of Aerial Parts of *N. Sintenisii* in Murine Melanoma B16F10 Cells

**Published:** 2018

**Authors:** Maryam Akaberi, Seyed Ahmad Emami, Mohsen Vatani, Zahra Tayarani-Najaran

**Affiliations:** a *Department of Pharmacognosy, School of Pharmacy, Mashhad University of Medical Sciences, Mashhad, Iran. *; b *Student Research Committee, Mashhad University of Medical Sciences, Mashhad, Iran. *; c *Department of Pharmacodynamics and Toxicology, School of Pharmacy, Mashhad University of Medical Sciences, Mashhad, Iran.*

**Keywords:** Melanogenesis, *Nepeta sintenisii*, Lamiaceae, Tyrosinase, Microphthalmia-associated transcription factor, MTT

## Abstract

*Nepeta* (Lamiaceae) is an important genus with beneficial medicinal properties. *N. sintenisii *Bornm. has been used in folk medicine of Iran to cure various diseases. We investigated the anti-melanogenesis effects of *n*-hexane, MeOH, CH_2_Cl_2_, *n*-BuOH, EtOAc, and H_2_O extracts isolated from the plant in B16 melanoma cells. Various assays including cytotoxicity, mushroom tyrosinase inhibition, inhibition of cellular tyrosinase, melanin content, the amount of reactive oxygen species and western blotting were done to assess the plant activities on melanogenesis inhibition. All extracts of *N. sintenisii* could significantly reduce both tyrosinase activity and the cellular melanin content. Reactive oxygen species were also significantly decreased following the treatment of cell with *n*-BuOH and EtOAc extracts with no cytotoxicity. The plant significantly decreased the amount of microphthalmia-associated transcription factor proteins. Collectively, *N. sintenisii* inhibited melanin synthesis and tyrosinase activity in B16 melanoma cells with no cytotoxic effects. Hence, it might merit further investigations for elucidation of anti-hyperpigmentation agents.

## Introduction

Melanin which is synthesized in melanosomes of melanocytes plays an important role in skin pigmentation in mammals (1). Melanogenesis (melanin synthesis) is regulated bytyrosinase-related protein 1 (TRP-1) and tyrosinase-related protein 2 (TRP-2) (2-4). The hydroxylation of tyrosine to 3,4-dihydroxyphenylalanine (DOPA) and hydroxylation of DOPA to DOPAquinone are the two initial steps of melanogenesis process catalyzed by tyrosinase (5). The alteration of DOPAchrome to 5, 6-dihydroxyindole- 2-carboxylic acid (DHICA) and the oxidation of DHICA to indole-5, 6-quinone-2-carboxylic acid are regulated by TRP-2 and TRP-1 respectively (2). Melanin has photoprotective effects against UV irradiation and is mediated by various signaling pathways (6, 7); however, a cAMP-dependent pathway is supposed to be the major pathway in independent stimulation of melanin production (8, 9). When the keratinocytes in the skin expose to ultraviolet irradiation (UV), they catalyze the production and release of α-melanocyte-stimulating hormone (α-MSH) which binds to the melanocortin 1 receptor, thereby activating adenylatecyclase via G proteins and elevate the intracellular cAMP levels (1, 10 and 11). As a result, protein kinase A which increases the expression of microphthalmia-associated transcription factor (MITF) is activated by cAMP. Melanocyte pigmentation, proliferation, and survival are believed to be regulated by MITF (12, 13). MITF, after binding the promoter regions of the genes, mediates the transcriptional activation of tyrosinase, TRP-1, and TRP-2 genes (4, 12). On the other hand, several skin disorders, including melanoma, freckles, nevus, and age spots are caused by excessive melanin biosynthesis which could be the result of increased numbers of melanocytes or activity of melanogenic enzymes which in turn cause epidermal and dermal hyperpigmentation (14). As the melanogenesis process could be stopped by inhibition of tyrosinase activity, many tyrosinase inhibitors such as kojic acid and arbutin (Maeda and Fukuda, 1996) (15) have been applied in the treatment of hyperpigmentation related problems. Because many synthetic skin-whitening agents have adverse side effects, natural products have emerged as an alternative solution (16). 

*Nepeta* (Lamiaceae) with about 250 species worldwide is a genus of perennial or annual herbs distributed mostly in Asia, Europe, and North Africa (17). There 79 species present in Iran (18) of which 38 species are endemic to the country (19) In folk medicine, *Nepeta* species are widely used as expectorant, antispasmodic, antiseptic, diuretic, antiasthmatic, antitussive, andfebrifuge (20, 21). *Nepeta sintenisii *Bornm. is a herbaceous wild plant growing wild in Iran, and central Asia (22, 23).

Natural products are valuable sources for discovering new drugs. The antioxidant activities of *Nepeta* spp. are well accepted (24, 25). This antioxidant property is attributed to the presence of various phytochemicals especially phenolic compounds (26, 27). On the other hand, it is believed that naturally occurring antioxidants including phenolic compounds have exerted anti-melanogenesis activities. Thus, in a screening program we evaluated the anti-melanogenesis activities of some plants with high antioxidant activities including *N. sintenisii*. In this study, the anti-melanogenesis effect of *N. sintenisii* on cultured murine B16F10 melanoma cells has been studied. MeOH extract of *N. sintenisii* was standardized by HPLC according to rosmarinic acid.

## Experimental

*Chemicals and Reagents*

The reagents and materials were obtained from different companies. Resazurin and rosmarinic acid from Sigma (Saint Louis, MO, USA) and trifluoroacetic acid (TFA, International Laboratory, USA); RPMI-1640 and FCS from PAA; β-actin and anti-rabbit IgG and HRP linked antibody from CellSignaling Technology (Boston, USA); Tyrosinase (H-109) and MITF (H-50) rabbit polyclonal antibody from Santa Cruz Biotechnology, Inc. (Dallas, Texas 75220 USA); Western blotting detection reagent from Bio-RaD (USA); α-melanocyte stimulating hormone,3,4-dihydroxy-L-phenylalanine, mushroom tyrosinase, phosphatase inhibitor cocktail, protease inhibitor cocktail, phenylmethylsulfonyl fluoride and QuantiPro BCA Assay Kit from Sigma (Steinheim, Germany). All solvents as analytical grade were purchased from Dr. Mojallali Lab. (Tehran, Iran).Water was purified using a Milli-Q water system (Millipore; Bedford, MA, USA).

*Plant materials*

The aerial parts of *N. sintenisii* were collected in July 2013 at altitude 1750 m from the National Park of Golestan, situated in Golestan province, Iran. Voucher specimens were identified in the Herbarium of Faculty of Pharmacy, Mashhad Medical University, and deposited under Accession No. 13061. The air dried aerial parts were powdered and then applied in the following process.

*Plant material extraction*

Plant materials were extracted with pure MeOH for 24 h by the percolation method at room temperature. To afford a crude MeOH extract, the filtered whole MeOH extract was concentrated in reduced pressure at 40-45 °C. This extract was then suspended in 95% MeOH and successively partitioned between *n*-hexane, MeOH, CH_2_Cl_2_, *n*-BuOH, EtOAc, and H_2_O. The extracts were evaporated under reduced pressure. All plant extracts were dissolved in DMSO in a concentration of 50 mg/mL and then stored at -20 °C until use.

*Cell culture*

B16F10 melanoma cells, purchased from Pasteur Institute of Iran, were maintained as a monolayer culture in Roswell Park Memorial Institute medium (RPMI 1640; PAA, Austria) supplemented with 10% heat-inactivated fetal bovine serum (FBS; Gibco, USA), 100 units/mL of penicillin, 0.1 mg/mL of streptomycin (antibiotic-antimycotic; PAA, Austria) in a humidified atmosphere containing 5% CO_2_ in air at 37 °C. The culture medium was changed every 2 d.

*Cell viability assay*

Conversion of resazurin (Sigma, MO, USA) to resorufin is a reduction reaction indicating the health of cells in which the reduction occurs in the cytosol of the live cells. While resazurin is non-toxic, blue in color and virtually non-fluorescent but resorufin is highly fluorescent and red in color. Resazurin continuously convert to resorufin in viable cells. This change increases the total fluorescence and color of the media surrounding cells (28).

B16F10 murine melanoma cells were seeded onto 96-well dishes at a density of 10^4^ cells per well. Then resazurin (14 mg/dL; 20 µL) was added to each well after 48 h incubation with 50 µg/mL of each extracts. The absorbance was measured at 570 and 600 nm after 4 h incubation at 37 °C. Cells incubated with 0.05% of DMSO and kojic acid were used as vehicle and positive controls, respectively. Culture medium was used as background. All experiments were performed in triplicate. 

*Melanin content determination*

The method described by Hosoi *et al.* was used for measuring the melanin content by some modifications (29). B16F10 melanoma cells were seeded at a density of 5 × 10^4^ cells per well in 96-well culture plates and cultivated by the method described above. The concentration of 50 µg/mL of each extract was added to the medium and incubated further for 24 h. Then, the medium was removed, and the cells were washed twice with phosphate-buffered saline (PBS) and harvested by trypsinization. The harvested cells were pelleted and the cell membrane was dissolved in Triton X-100. Then, the purified melanin was dissolved in 2M NaOH for 30 min at 100 °C. The absorbance was measured at 405 nm and melanin content was compared with control untreated cells. Cells incubated with 0.05% of DMSO and kojic acid were used as vehicle and positive controls, respectively. Culture medium was used as background.

*Mushroom tyrosinase activity assay*

In a 96-well plate 10 µL of each sample extracts (50 µg/mL) were mixed with 160 µL of 5 mM L-DOPA (in 100 mM sodium phosphate buffer pH 6.8) was added to the wells plus 20 µL of mushroom tyrosinase and shacked for 5 min. Kojic acid was used as positive control and cell containing medium as negative control. The plate incubated for 30 min at 37 °C and the amount of dopachrome produced in this mixture was measured by spectrophotometer at 490 nm by ELISA Reader.

*Cellular tyrosinase activity assay*

Tyrosinase activity was analyzed by spectrophotometry following the oxidation of DOPA to DOPAchrome. After B16F10 melanoma cells (5 × 10^4^ cells/well) were seeded in 96-well plate and incubated for 24 h, they were treated with concentration of 50 µg/mL of the extracts. After trypsinization, the harvested cells were pelleted and washed with PBS. Then, pelleted cells lysed with 100 µL sodium phosphate buffer 100 mM (pH 6.8) containing 1% Triton X-100 and 0.1 mM PMSF. After 30 min, the lysates were centrifuged at 10,000 rpm for 20 min at 4 °C. The supernatant was transferred to 1 mL sterile microtube and kept in -80 °C. Hundred µL of this protein suspension as well as 100 µL of DOPA 5 mM was added to each well of a 96-well plate. After 2 h incubation, the absorbance was measured at 475 nm.

*Western blotting*

B16F10 melanoma cells were cultured in 25 cm^2^ flasks as described above. Cells were treated with all extracts at the concentration of 50 µg/mL for 24 h. The cells were then lysed in a buffer containing 50 mM Tris-HCl with pH 7.4, 2 mM EGTA, 1 mM phenylmethylsulfonyl fluoride, 10 mM β-Glycerophosphate, 1 mM sodium orthovanadate, 10 mM β-mercaptoethanoland 0.1% deoxycholic acid sodium salt (Western Blotting Protocol, BioRad, USA).

Proteins (50 µg) were resolved by 10% SDS-polyacrylamide gel electrophoresis and transferred electrophoretically to polyvinylidene difluoride membranes and then were blocked overnight in 5% skim milk in TBST (containing 20 mM Tris-HCl pH 7.4, 100 mM NaCl, and 0.1% Tween 20) buffer at 4 °C. After washing in TBST buffer, they were then incubated for 3 h with a primary antibody: rabbit anti-tyrosinase antibody (1:300), anti- MITF antibody (1:300). After incubation with an anti-rabbit IgG (1:2000) as a secondary antibody, the bands were detected using the ECL Prime Western Blotting Detection System (BioRaD, USA). Bands were scanned by GS-800, and band intensities were quantified by measuring optical densities with Quantity One software (Bio-Rad).

*Cellular ROS level determination*

About 5 × 10^4^ B16F10 melanoma cells were cultured in 96-well plate and treated with 50 µg/mL of the extracts and positive control for 24 h. Cell were exposed to 24 mM H_2_O_2_ at 37 °C for 30 min and 2ꞌ, 7ꞌ-dichlorofluorescein diacetate (DCFH-DA) was added to each well and incubated for the next 30 min. ROS was measured according to fluorescence intensities of DCF at excitation wavelength 504 nm and emission wavelength 524 nm using a Synergy H4 Hybrid Multi-Mode fluorescent Microplate Reader (BioTek, Winooski, USA).

*Quantitative analysis of rosmarinic acid by HPLC-DAD sample preparation*

MeOH extract of *N. sintenisii* was standardized by HPLC using rosmarinic acid. The analysis of rosmarinic acid was performed using reversed-phase HPLC with a linear gradient mobile phase of 50%-100% methanol in H_2_O including 0.05% trifluoroacetic acid (TFA) with a flow rate of 1.0 mL/min at room temperature. First of all, different concentrations (125, 250 and 500 µg/mL) of the standard (rosmarinic acid) were prepared for plotting the standard curve. Then, about 20 g of the dried MeOH extract was dissolved in 1 mL dimethylsolfoxide (DMSO) and filtered through a syringe filter (0.4 μm, Alltech, Deerfield, IL, USA). The sample and standards were loaded on a C18 column (Advanced Chromatography Technologies Limited, Aberdeen, Scotland). Injection volume was 10 μL and analysis was monitored at 254 nm. To validate the gradient method, a standard sample containing uracil, methyl p-hydroxy benzoate, ethyl p-hydroxy benzoate, and benzophenone was injected repeatedly until four thoroughly separated peaks were observed.

*Statistics*

Values were expressed as a mean ± standard deviation (SD) of three different experiments. To check for quantitative differences between the groups, analysis of variance (ANOVA) and the Dunnettꞌs Multiple Comparison tests were performed with GraphPad Prism 5.0.

## Results

*Effect of N.* *sintenisii extracts on melanin synthesis in B16F10 cells without cytotoxicity*

**Figure 1:**
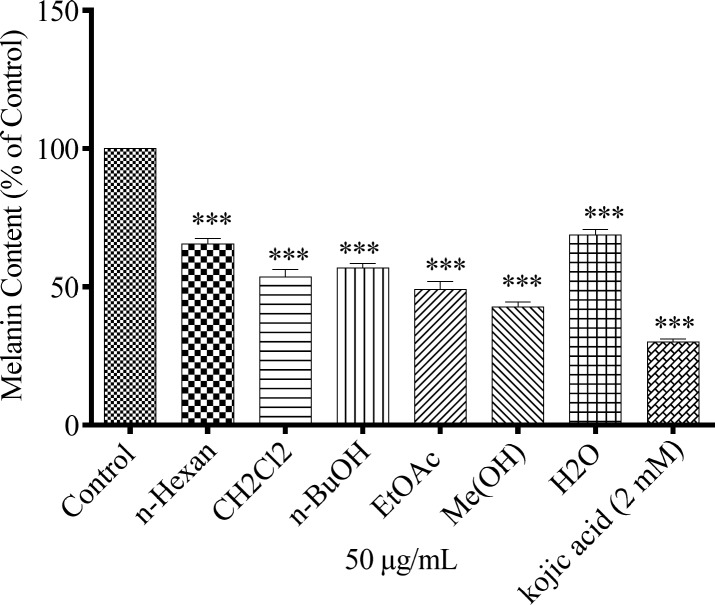
Effect of *N. sintenisii* on melanin content in B16F10 murine melanoma cells. B16F10 cells were incubated without (control) and with 50 µg/mL of different *N. sintenisii *extracts for 48 h. Melanin content was measured as described in ‘‘Materials and Methods.’’ As shown in Figure all extracts significantly decreased the melanin content in B16F10 cells. Results were expressed as percentages relative to control, and are presented as mean ± SD of triplicate samples. Statistically significant difference between extract-treated cells and control ^***^(*P *< 0.001

**Figure 2:**
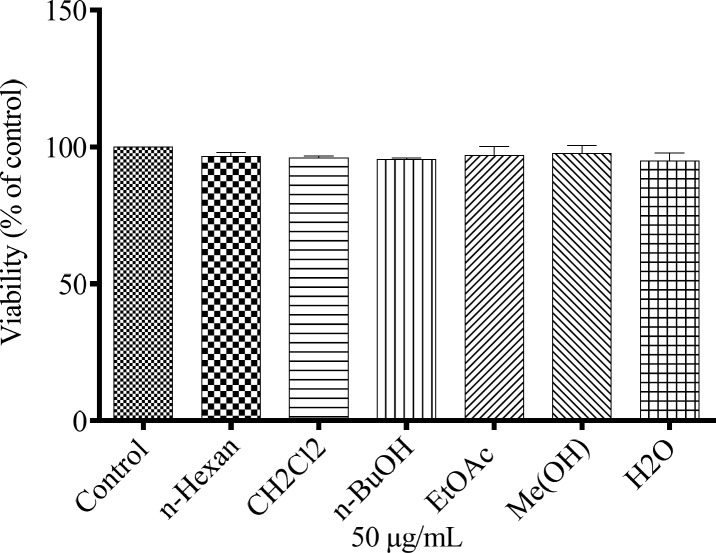
Cytotoxic effects of *N. sintenisii* extracts on murine melanoma cells. After incubation of B16F10 melanoma cells with concentration of 50 µg/mL of different extracts of *N. sintenisii* in a 96-well plate for 48 h, cell viability was determined by Resazurin assay. Percentage values in the treated cells were compared with respect to that in the control cells. Data are expressed as mean ± SD for triplicate samples

To study the effect of different extracts of *N. sintenisii* on melanin synthesis, the melanin content of the extract-treated B16F10 melanoma cells was quantified. The melanin contents were significantly decreased in the extract treated cells (Figure 1). In addition, investigation of the effect of *N. sintenisii *extracts on B16F10 melanoma cell proliferation showed that the extracts had no significant cytotoxic effect on B16F10 cells at the concentration used (Figure 2). These results indicate that *N. sintenisii *extract exerts anti-melanogenic effects on B16F10 melanoma cells without inducing cytotoxicity.

*Effect of N*. *sintenisii* *extracts on mushroom tyrosinase activity*

**Figure 3:**
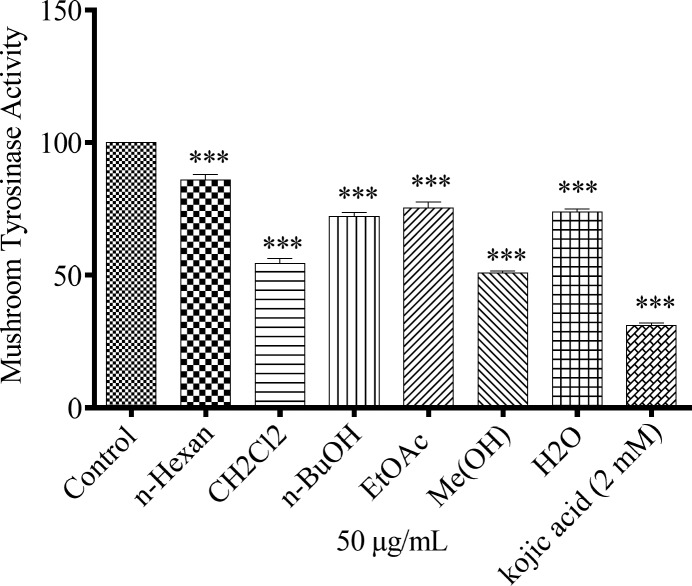
Effect of *N. sintenisii* extracts on mushroom tyrosinase in B16F10 murine cells. Concentration 50 µg/mL of different *N. sintenisii* extracts and 2 mM of Kojic acid were incubated with mushroom tyrosinase and L-DOPA at 37 °C. Mushroom tyrosinase activity was measured by the change in absorption at 475 nm. Results were expressed as percentages relative to control, and are presented as mean ± SD of triplicate samples. Statistically significant difference between extract-treated cells and control ^*^(*P *< 0.05), ^**^(*P *< 0.01) and ^***^(*P *< 0.001

To determine whether *N. sintenisii *extracts affect tyrosinase activity directly, we performed a mushroom tyrosinase assay using L-DOPA as substrate and mushroom tyrosinase as enzyme source. Tyrosinase is a key enzyme catalyzing the rate-limiting step in melanin biosynthesis. As shown in Figure 3, all extracts, especially CH_2_Cl_2_ and MeOH extracts of* N. sintenisii *exerted a significant inhibitory effect on L-DOPA oxidation by mushroom tyrosinase and significantly reduced the activity of mushroom tyrosinase. These results suggest that CH_2_Cl_2_ and MeOH extracts of *N. sintenisii* displayed the most inhibitory activity on mushroom tyrosinase activity and there was no significant difference between CH_2_Cl_2_ and MeOH extracts with kojic acid.

*Inhibitory effect of N. sintenisii extracts on* *B16F10 melanoma cellular tyrosinase activity*

**Figure 4:**
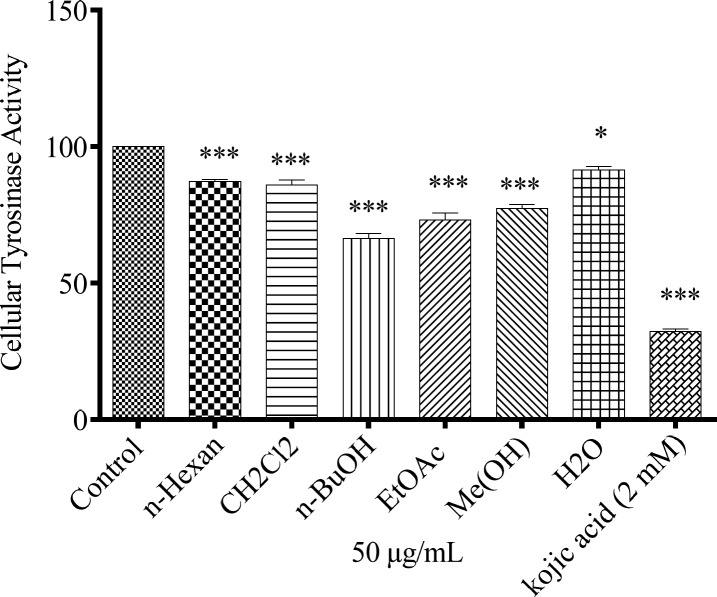
Effect of *N. sintenisii* extracts on cellular tyrosinase in B16F10 murine melanoma cells. After incubation of B16F10 melanoma cells with concentration 50 µg/mL of different *N. sintenisii* extracts for 48 h, cellular tyrosinase activity was assessed as described in ‘‘Materials and Methods’’ Results were expressed as percentages relative to control, and are presented as mean ± SD of triplicate samples. Statistically significant difference between extract-treated cells and control ^**^(*P *< 0.01) and ^***^(*P *< 0.001

The anti-melanogenesis effect of different extracts of *N. sintenisii* on cellular tyrosinase activity was assessed. As shown in Figure 4, suppression of tyrosinase activity in the cultured B16F10 melanoma cells was occurred after treatment of the cells with 50 µg/mL of all extracts with different ratios.

*Effect of N. sintenisii extracts on the Cellular ROS level*

**Figure 5:**
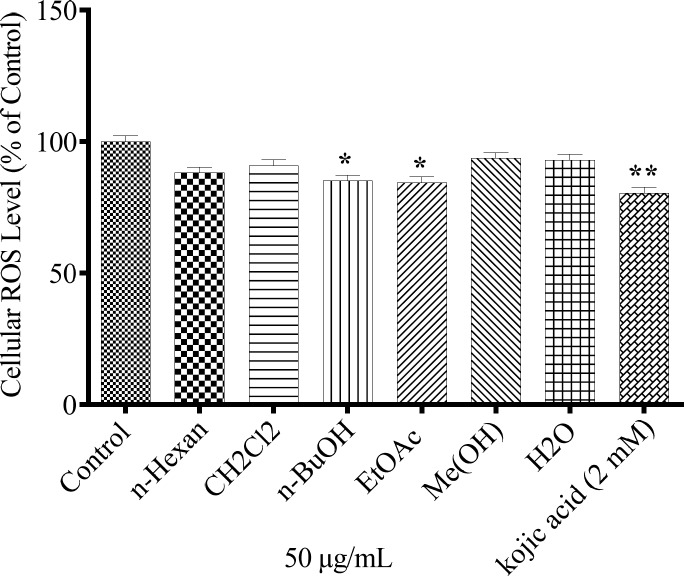
Antioxidanteffect of *N. sintenisii* extracts andcellular ROS levelin B16F10 murine melanoma cells. The cells were treated with concentration 50 µg/mL of different *N. sintenisii *extracts or Kojic acid (2.0 mM) for 24 h and then the ROS content was measured by the DCF-DA assay. Results were expressed as percentages relative to control, and are presented as mean ± SD of triplicate samples. Statistically significant difference between extract-treated cells and control ^**^(*P *< 0.01) and ^***^(*P *< 0.001

The antioxidant capacity of different extracts was measured with respect to intracellular ROS levels. 24 mM H_2_O_2_ was exposed to cells pretreated with plant extracts. As shown in Figure 5, only *n*-BuOH and EtAOc extracts could significantly suppress the oxidative stress induced by H_2_O_2_ in the cells.

*The level of tyrosinase and MITF protein by N*. *sintenisii* *extracts*

**Figure 6:**
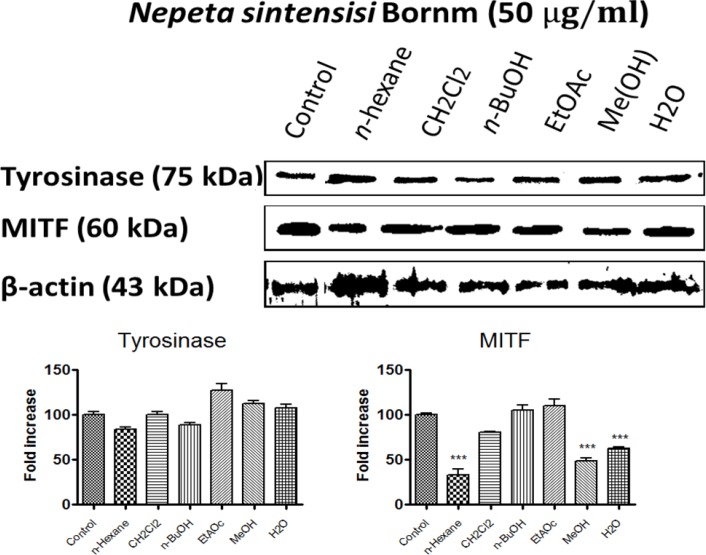
Effect of *N. sintenisii* on tyrosinase and MITF protein level in B16F10 murine melanoma cells. B16F10 melanoma cells were treated with 50 µg/mL of different *N. sintenisii* extracts for 48 h. The loading control was β-actin antibody. The relative intensities of tyrosinase and MITF protein compared with the β-actin using Quantity One software

To determine the tyrosinase protein level in the cells treated with *N. sintenisii *extracts, we performed Western blot analysis (Figure 6) and also the intracellular effect of different extracts on melanogenic related proteins such as tyrosinase and MITF as an indicator of melanogenesis was evaluated. As shown in Figure 6, while tyrosinase protein levels were not decreased significantly by all extracts of *N. sintenisii, *MITF was significantly decreased following treatment with *n*-hexane, MeOH, and H_2_O extracts. β-Actin was used as internal control. 

These results suggest that the anti-melanogenesis effect of the mentioned extracts on B16F10 cells is not associated with the down regulation of tyrosinase; the most important enzyme in melanogenesis.

*Quantitative analysis of rosmarinic acid by HPLC-DAD sample preparation*

**Figure 7:**
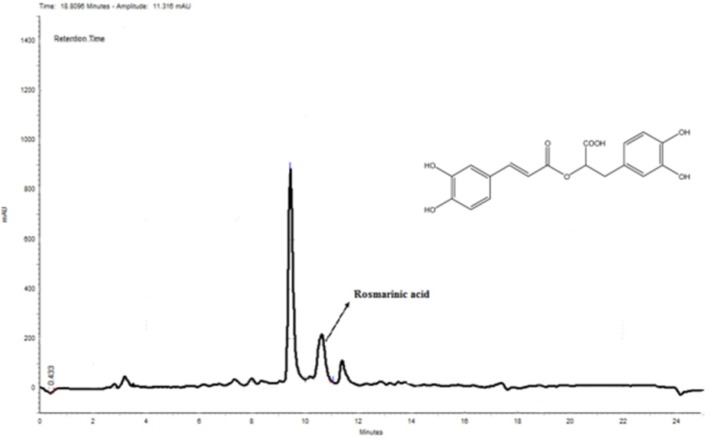
HPLC chromatogram of MeOH extract. Rosmarinic acid was used as a standard. According to the obtained standard curve (Y = 0.0182X - 0.0002) (data is not shown), the amount of rosmarinic acid was 10.35 mg in 1000 mg of the extract

Rosmarinic acid is a caffeic acid ester of salvianic acid A (3,4-dihydroxyphenyllactic acid) with antioxidant properties found in a variety of plants. Rosmarinic acid was first isolated from rosemary (*Rosmarinus officinalis*). The chromatogram of MeOH extract is shown in Figure 7. The rosmarinic acid was spiked in the sample and its concentration was determined using the obtained standard curve (Y = 0.0182X - 0.0002). There are 10.35 mg rosmarinic acid per 1000 mg of the extract; thus, the percentage of rosmarinic acid is 1.035 (W/W) in the sample. As it can be seen in Figure 7, there is also another major compound before the peak of rosmarinic acid which can be another bioactive component of this extract.

## Discussion

Melanoma, the deadliest form of skin cancer, is one of the most challenging human cancers. Recently, the demand for natural anti-melanogenic agents, both for the treatment of cancers and for cosmetics has increased worldwide. In addition, ageing is one of the most important factors which increase melanin-rich spots on the skin, in which melanogenesis plays an important role. Therefore, regulating melanogenesis is an interesting field of research and we would like to search for new agents for this purpose. Because of the side effects of synthetic drugs, such as 3,4-dihydroxyacetophenone (30), phospholipase D1 (31, 32), and phospholipase D2 (33, 34) we have focused on finding new agents regulating melanogenesis from natural resources.

In this study, we investigated whether different extracts of *N. sintenisii *had have an anti-hyperpigmentation effect on B16F10 melanoma cells. It was found that all extracts significantly down-regulated melanin synthesis without significant cytotoxicity. Generally, MeOH extract of *N. sintenisii* was the most active extract in all tests except in cellular tyrosinase activity test in which butanol extract was the most active one; thus MeOH extract was standardized by HPLC. MeOH extract considerably inhibited melanogenesis with no cytotoxicity (Figures 1 and 2). The observed activity for MeOH extract can be attributed to rosmarinic acid or another major component present in this extract. After 48 h of treatment with the extracts, B16F10 cells were still completely viable, which is an important point in using plants for cosmetic or medical purposes. The anti-melanogenesis effect of potent extracts without any cytotoxicity is an encouraging factor for developing natural anti-melanogenesis agent. 

Melanin synthesis occurs predominantly in the lysosome-like structures of melanocytes known as melanosomes by which melanin is packaged and delivered to the keratinocytes. There are two types of melanin, pheomelanin, and eumelanin which are different in color, size, shape, and the packing of their granules. Both pheomelanin and eumelanin are produced from tyrosine by tyrosinase-dependent pathway. Melanogenesis is believed to be regulated by the tyrosinase gene family, including tyrosinase, TRP-1, and TRP-2 (12, 35). Melanin production is supposed to be correlated with the expression level and the catalytic activity of tyrosinase.

To investigate the mechanism of *N. sintenisii *extract in melanogenesis inhibition, first we investigated the inhibitory activity of *N. sintenisii *extracts directly in cell-free assay systems, using mushroom tyrosinase as enzyme source and kojic acid as positive control, since it has inhibitory activity on melanin synthesis in melanoma cells. All extracts of *N. sintenisii *had inhibitory effect on tyrosinase activityand significantly decreased the mushroom tyrosinase and cellular tyrosinase activity after 24 h (Figures 3 and 4). Moreover, there was a significant decrease in ROS content in the cells treated with *n*-BuOH and EtAOc extracts showing the antioxidant capacity of these extracts. It seems that the inhibitory effect on tyrosinase activity does not correlate with antioxidant capacity of these extracts.

Microphthalmia-associated transcription factor (MITF), one of the most important nuclear transcription factors, regulates melanogenesis by activation of tyrosinase, TRP1 and TRP2 transcription (36). Studies show that in addition to regulating melanogenesis, MITF also plays an important role in the regulation of melanocyte development, survival, and tumorigenesis as well as progression of melanoma. Recently, MITF has been studied as a potential molecular target for melanoma therapy. The *n*-butanol and MeOH extracts of *N. sintenisii* considerably decreased the amount of tyrosinase and microphthalmia-associated transcription factor proteins. Many signal transduction pathways have been found to balance melanin production such as mitogen-activated protein kinase pathways, especially the extracellular signal-regulated kinase (ERK) 1/2 pathway, that are probable pathways for MITF regulation (37-42). When MITF is phosphorylated at serine 73 by ERK, significant degradation occurs leading to ubiquitin-dependent proteasomal degradation. In the present study, tyrosinase protein levels were not decreased significantly by all extracts of *N. sintenisii *but MITF was significantly decreased following the treatment with *n*-hexane, MeOH, and H_2_O extracts. Decrease in MITF level obviously confirms the inhibitory effect on melanin production by the plant. Since changes in protein level in the cells are time dependent, no change in the tyrosinase level may be associated with the time that assay was performed.

Although in several studies the antioxidant activities of *Nepeta* spp. are reported (24, 25) there is no investigation on the antioxidant property of the extracts from this species namely *N. sintenisii*. There is only one recent study about the antioxidant activity of *N. sintenissi *which investigated the essential oil of this plant (43). Thus we evaluated the antioxidant activity of this plant for the first time. Our observations show that different extracts of *N. sintenisii* has strong antioxidant activity to some extent. In addition, up to now no study has reported the constituents of *N. sintenisii*. To examine the bioactive components of these extracts further evaluations seem necessary.

Taken together, the present study revealed that *N. sintenisii *has significant and anti-melanogenesis activities which does not correlate with ROS scavenging activity of the plant. *N. sintenisii *extracts inhibited cellular melanin biosynthesis and tyrosinase activity in B16F10 murine melanoma cells and decreased MITF level. These results indicate that *N. sintenisii *extracts have significant inhibitory effects on melanogenesis and can be used as a therapeutic treatment for skin hyperpigmentation diseases.
